# Improvement of reversible compressibility of ultralight carbon nanotube/carboxymethyl cellulose materials through hydrophobic surface treatment

**DOI:** 10.1080/14686996.2025.2580919

**Published:** 2025-11-06

**Authors:** Reo Yanagi, Hitomi Shimamura, Kenta Ono, Junko Hieda, Tomonaga Ueno

**Affiliations:** Department of Chemical Systems Engineering, Graduate School of Engineering, Nagoya University, Nagoya, Japan

**Keywords:** Ultralight, carbon nanotubes, carboxymethyl cellulose, composites, hydrophobization, compressibility

## Abstract

The mechanical properties of ultralight materials are influenced by their constituent materials, internal porous structures, and surface states. The surface chemical state is particularly crucial for materials composed of hydrophilic polymers such as cellulose, where interactions with ambient water are significant. In this study, we report that the reversible compressibility of ultralight carbon nanotube (CNT)/carboxymethyl cellulose (CMC) materials can be enhanced by hydrophobic surface treatment with silane coupling agents. We examined the treatment conditions for hydrophobization and investigated their impact on the microstructure and surface chemical properties of the ultralight material. The hydrophobized ultralight CNT/CMC materials with a bulk density of 1.6 mg/cm^3^ demonstrated superior reversible compressibility, with a 65% recovery rate even under high humidity conditions (80% RH).

## IMPACT STATEMENT

This study investigated the effect of surface functional groups on the reversible compressibility of ultralight materials and succeeded in producing samples with reversible compressibility even under high humidity.

## GRAPHICAL ABSTRACT

**Figure:**
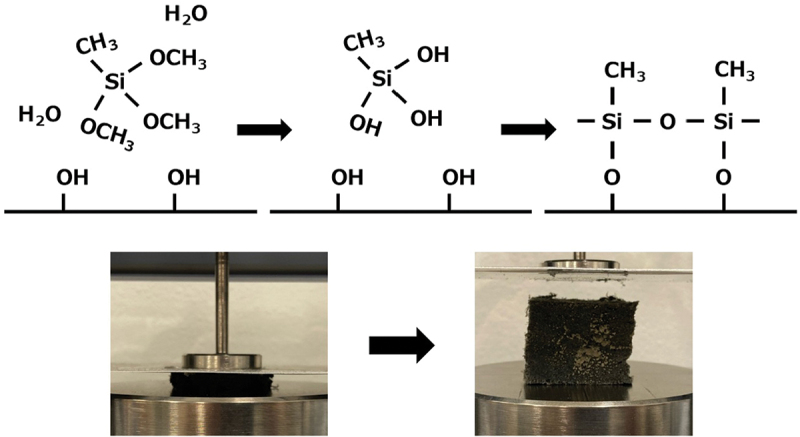

## 1. Introduction

Ultralight materials are materials with a bulk density of less than 10 mg/cm^3^, with porous materials such as aerogels [1,2] and microlattices [3] being prime examples. Recently, research on ultralight materials with bulk densities close to or even lower than that of air (about 1.2 mg/cm^3^ at room temperature) has been actively conducted [4,5]. Ultralight materials made from nanocarbon, such as carbon nanotubes (CNTs) and graphene, are gaining attention as ultralight functional materials due to their lightweight nature and excellent mechanical [6], thermal [7], and electrical properties [8]. Specifically, they have potential applications in ultralight wearable sensors [9], sound-absorbing materials [10], and electromagnetic wave shielding and absorbing materials [11]. However, their low density poses a challenge due to the resulting low mechanical properties.

The mechanical properties of ultralight materials are significantly influenced by the internal porous structures and the surface condition of the walls that compose these porous structures. Ultralight materials compress as their porous structures collapse under compression, and their ability to regain structure gives them excellent reversible compressibility [12]. These materials typically have a porosity exceeding 99%, resulting in an extremely large specific surface area [10]. During compression, many of the walls that form these pores come into contact, and consequently, the surface condition of these walls significantly affects the reversible compressibility of the materials. Enhancing reversibility is particularly important for practical applications that require repeated mechanical deformation [13,14]. While many reports have focused on enhancing the reversible compressibility of ultralight materials by controlling their internal porous structures [12,15], studies exploring the relationship between surface conditions and reversible compressibility have been limited.

The hydrophilicity of the surface is one of the critical surface conditions that significantly affect the mechanical properties of ultralight materials. Cellulose-based polymers are known for their ability to disperse nanocarbons well in water, leading to extensive research on ultralight nanocarbon/cellulose composite materials [16]. In particular, carboxymethyl cellulose (CMC) is non-covalently adsorbed onto the surface of carbon nanotubes (CNTs) via CH–π interactions [17]. The carboxymethyl groups of CMC induce electrostatic repulsion through Coulombic forces, resulting in a stable aqueous dispersion while preserving the intrinsic CNT framework. Compared with common non-covalent dispersants such as sodium dodecylbenzene sulfonate (NaDDBS) and octenyl-succinic-anhydride (OSA) – modified starch, CMC has been shown to produce the most stable CNT suspensions [18]. By these characteristics, the use of CMC enables the facile fabrication of ultralight yet mechanically robust CNT frameworks with uniform dispersion and strong inter-fiber connectivity. However, these materials exhibit hydrophilicity due to the abundant hydroxyl groups in cellulose, limiting their mechanical properties in high-humidity environments [19]. It is known that porous materials exhibiting hydrophilicity due to surface hydroxyl groups demonstrate reduced mechanical properties owing to their hygroscopic nature.

Surface hydrophobization is an effective strategy to enhance the mechanical performance of such ultralight materials. Conventional methods, such as carbonization and plasma treatment [20,21], can improve surface properties but often involve cutting of polymer main chains and require specialized equipment. In contrast, gas-phase silane coupling offers a simpler and lower-cost approach [22] and has been shown to improve the compressive recovery of bio-based aerogels [23]. Furthermore, regarding surface functionalization using silane coupling, while there is a method involving liquid-phase silanization after UV/O_3_ pretreatment [24], for materials utilizing cellulose – which is particularly rich in hydroxyl groups – gas-phase hydrophobic modification enables simpler and lower-cost silane modification. However, the effects of gas-phase silane hydrophobization on CNT/Cellulose composite materials – particularly its influence on compression recovery under varying humidity – have yet to be systematically investigated [25].

In this study, we investigated the effects of hydrophobization treatment on the reversible compressibility of ultralight CNT/carboxymethyl cellulose (CMC) materials by modifying the surface state using silane coupling agents. Ultralight CNT/CMC materials with a bulk density of 1.3 mg/cm^3^ were fabricated using a freeze-drying method, and the surface was hydrophobized using two types of silane coupling agents – trimethoxy(methyl)silane (MTMS) and (tridecafluoro-1,1,2,2-tetrahydrooctyl)trimethoxysilane (FAS13). We evaluated the changes in microstructure and surface functional groups due to the hydrophobization treatment and examined the impact on reversible compressibility. The hydrophobization treatment enabled the materials to exhibit high reversible compressibility even under high-humidity environments.

## 2. Experimental methods

### 2.1. Materials

CNTs (eDIPS EC 2.0, Meijo Nano Carbon Co., Ltd., Aichi, Japan) with a diameter of 1–3 nm and a length of 1 µm or more were used. CMC with a weight-average molecular weight of 100,000–110,000 and Na content of 6.5–8.5% (Fujifilm Wako Pure Chemical Corporation) were used. The silane coupling agents used were trimethoxy(methyl)silane (Tokyo Chemical Industry Co., Ltd., hereafter referred to as MTMS) and (tridecafluoro-1,1,2,2-tetrahydrooctyl)trimethoxysilane (Gelest Inc., hereafter referred to as FAS13).

### 2.2. Preparation of ultralight CNT/CMC materials

**Figure 1.**
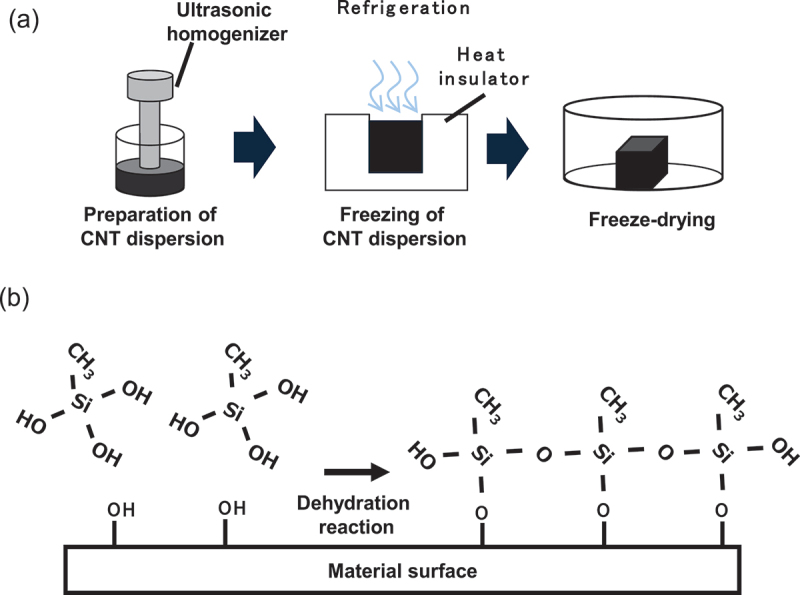
Schematic illustration of (a) the preparation process of the ultralight CNT/CMC materials and (b) the hydrophobization process using silane coupling agent.

Figure 1(a) schematically illustrates the preparation of ultralight CNT/CMC materials. CNT and CMC components were added to distilled water containing 1% ethanol. The sample composition was adjusted to 1.3 g/L to fabricate samples with a bulk density of 1.3 mg/cm^3^. The CNT mass ratio was 10%. The dispersions were ultrasonically agitated for 12 minutes using an ultrasonic homogenizer (Sonifier 450, Emerson Japan Ltd., Tokyo, Japan). These dispersions were then cooled at 5 °C for 30 minutes in a low-temperature water bath (LTB 125α, As One Corporation, Osaka, Japan) and subsequently frozen at −80 °C for 4 hours in an ultra-low temperature bath (VT-78, Nippon Freezer Co., Ltd., Tokyo, Japan). The dispersions were uniaxially frozen by covering the sides and bottom with a thermal-insulating material. The ice that formed in the sample during freezing created the pore structure of the sample after fabrication. Finally, the dispersions were freeze-dried using an FDU-12AS freeze dryer (As One Corporation, Osaka, Japan). Through this process, ultralight CNT/CMC materials were fabricated, each with dimensions of 40 mm × 40 mm × 40 mm.

### 2.3. Hydrophobization using silane coupling agent

Figure 1(b) schematically illustrates the process of hydrophobization using a silane coupling agent. The fabricated materials were placed in a PFA container (PF-180, As One Corporation, Osaka, Japan) together with each silane coupling agent and sealed. The container was subsequently placed in a constant temperature oven (EO-300V, As One Corporation, Osaka, Japan) and heated at the specified temperature for the specified time to proceed with the silane coupling reaction and achieve hydrophobization.

### 2.4. Materials characterization

The microstructure of the prepared samples was observed using field emission-scannning electron microscopy (FE-SEM, S4800, Hitachi High-Tech Corporation, Tokyo, Japan). The pore area and fiber diameter in the SEM images were quantified using ImageJ software. The pore area was determined from binarized SEM images, and the average fiber diameter was obtained by measuring ten randomly selected fibers in each image. The quantification of elemental concentrations on the material’s surface was performed using an X-ray detector (X-Max, Horiba Ltd., Kyoto, Japan) with energy-dispersive X-ray spectroscopy (EDX). During EDX analysis, the acceleration voltage was set to 15 kV and the emission current to 10 mA.

FTIR (FT/IR-4100, JASCO Corporation, Tokyo, Japan) was performed by the attenuated total reflection method in air over a measurement range of 500–4000 cm^−1^, with a resolution of 4 cm^−1^, and 16 cumulative scans. X-ray photoelectron spectroscopy (XPS) measurements were conducted using an ESCALAB 250Xi instrument from Thermo Scientific K.K., Tokyo, Japan. The spectral measurements were acquired using a monochromatic Al Κα X-ray source, with an irradiation area diameter of 500 μm.

We examined the mechanical properties by compression tests using a precision universal/tensile tester (AGS-5kNX, Shimadzu Corporation, Kyoto, Japan). The elastic modulus at 20% strain was measured from the obtained stress – strain curves. The recovery rates were measured after compressing to 80% strain (see Figure S1 for detailed measurement method). Each sample was compressed once at a crosshead speed of 1 mm/min, with the load applied perpendicular to the freezing direction [12].

## 3. Results and discussion

### 3.1. Samples before hydrophobization

**Figure 2.**
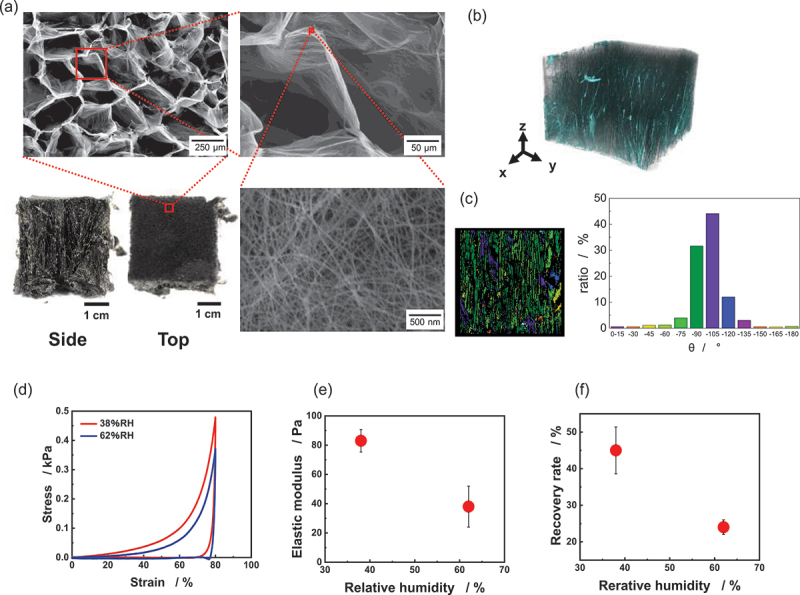
Microstructure and mechanical properties of ultralight materials before hydrophobization. (a) side and top views, and SEM images of the top surface. (b) X-ray CT image of a sample with a CNT mass ratio of 20% (adapted from [12]). (c) Cross-sectional view of the y-z plane and the orientation distribution. (d) Stress-strain curves, (e) elastic modulus, and (f) recovery rate measured under different relative humidity conditions (38% RH and 62% RH).

Figure 2(a) shows the side and top views and the SEM images of the ultralight CNT/CMC materials before hydrophobization treatment. The ultralight material has a honeycomb-like porous structure on the top surface. During the freezing process of the dispersion, all sides except the top are insulated, allowing ice crystals to grow from the top. At the freezing temperature of −80 °C, ice crystals form a hexagonal structure and grow as hexagonal columns. These ice crystals are removed through freeze-drying, resulting in the formation of honeycomb-like pores. The walls of these pores are composed of fibers consisting of CNTs and CMC. The average area of a single pore is 23,521 ± 12,385 µm^2^, with fiber diameters of 26 ± 6.2 nm. Figure 2(b,c) present X-ray CT images, cross-sectional views, and structural orientation distributions of samples prepared at a bulk density of 5 mg/cm^3^. To enhance detection accuracy, Pt nanoparticles were deposited on the CNT surfaces. The results indicate that the fabricated ultralight CNT/CMC material exhibits a highly oriented structure due to uniaxial freezing.

The fabricated ultralight materials exhibit hygroscopic properties due to the abundance of OH groups in the CMC. The excellent dispersibility of CNTs by CMC enables the formation of an ultralight yet mechanically robust framework; however, because CMC is highly hygroscopic, the mechanical characteristics of the material are strongly influenced by the relative humidity of the environment. Figure 2(d) presents the stress-strain curves of the untreated CNT/CMC materials under different relative humidity conditions (38% RH and 62% RH). Compression perpendicular to the freezing direction caused the pores to collapse before the honeycomb buckled, so no distinct yield point was observed up to 80% strain [12]. The stress values are consistently higher across the entire strain range at 38% RH compared to 62% RH. At 38% RH, the elastic modulus is 83 ± 7.7 Pa, whereas it decreases to 38 ± 14.0 Pa at 62% RH (Figure 2(e)). Furthermore, the recovery rate at 38% RH is 45 ± 6.4%, while it drops to 24 ± 2.0% at 62% RH (Figure 2(f)). The hydroxyl groups on the material surface facilitate water adsorption from the air. When CMC adsorbs water, intermolecular forces between water molecules cause the material to aggregate, resulting in reduced stress during compression. Additionally, this aggregation force diminishes the restoring force within the material after compression.

### 3.2. Samples after hydrophobization

Hydrophobization of the ultralight CNT/CMC materials was performed using silane coupling agents. Samples were prepared by varying the amount of MTMS used in the reaction: 0.98, 9.8, 19.6 and 29.4 mmol/gCMC. The samples were labeled as CCM (M (in case of FAS13, it is referred to as F), Quantity of MTMS (mmol/gCMC), Heating temperature (°C), Heating time (h)). For comparison, a sample subjected only to heat treatment without hydrophobization was prepared and labeled as CCM(N).

Table 1 shows the changes in mass and bulk density of each sample after hydrophobization. The hydrophobization process resulted in an increase in both mass and bulk densities. As the quantity of MTMS used increased, the mass increase rate increased. The mass decrease in CCM(N) can be attributed to the removal of adsorbed moisture through heating. The slight increase in density for CCM(N) is due to minor shrinkage of the material during the heating process.

**Table 1.** Mass and bulk density changes of each sample by hydrophobization.

Sample name	Weight increase rate (%)	Bulk density (mg/cm^3^)
CCM(N)	−8.5 ± 4.1	1.4 ± 0.05
CCM (M0.98, 200, 3)	0.2 ± 2.4	1.6 ± 0.08
CCM (M9.8, 200, 3)	4.2 ± 3.6	1.6 ± 0.08
CCM (M19.6, 200, 3)	6.0 ± 3.9	1.7 ± 0.08
CCM (M29.4, 200, 3)	6.8 ± 2.3	1.7 ± 0.05

**Figure 3.**
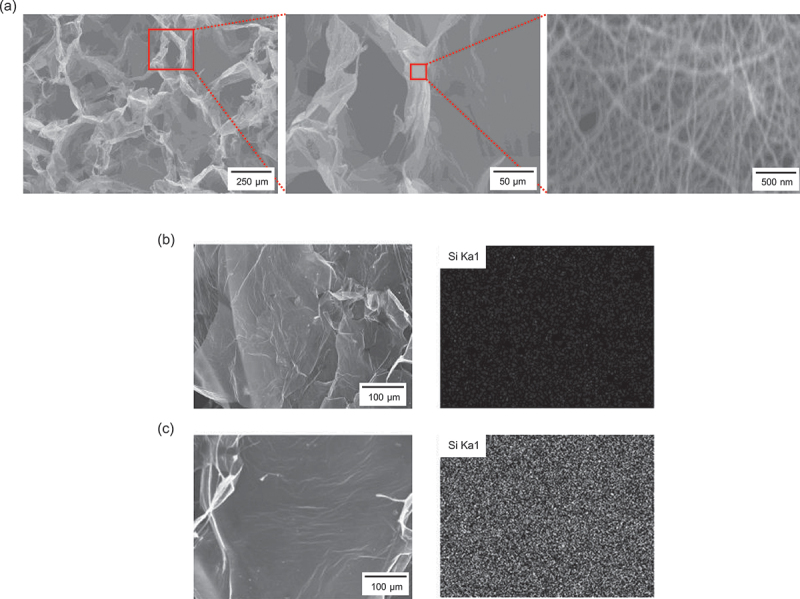
Microstructure of the ultralight CNT/CMC material. (a) SEM images of the surface after hydrophobization. EDX mapping for the Si element (b) before and (c) after hydrophobization.

Figure 3(a) presents SEM images of the ultralight CNT/CMC materials (CCM (M19.6, 200, 3)) after hydrophobization. The microstructure observed is similar to those before hydrophobization (see Figure S3 for other samples). The average area of a single pore was 23,824 ± 17,637 µm^2^, with fiber diameters of 28 ± 7.0 nm. Figure 3(b,c) show EDX mapping for the Si element in CCM(N) and CCM (M19.6, 200, 3), respectively. The mapping of CCM (M19.6, 200, 3) reveals a uniform distribution of Si elements on the surface. EDX analysis indicated a surface Si concentration of 0% for CCM(N) and 4% for CCM (M19.6, 200, 3).

**Figure 4.**
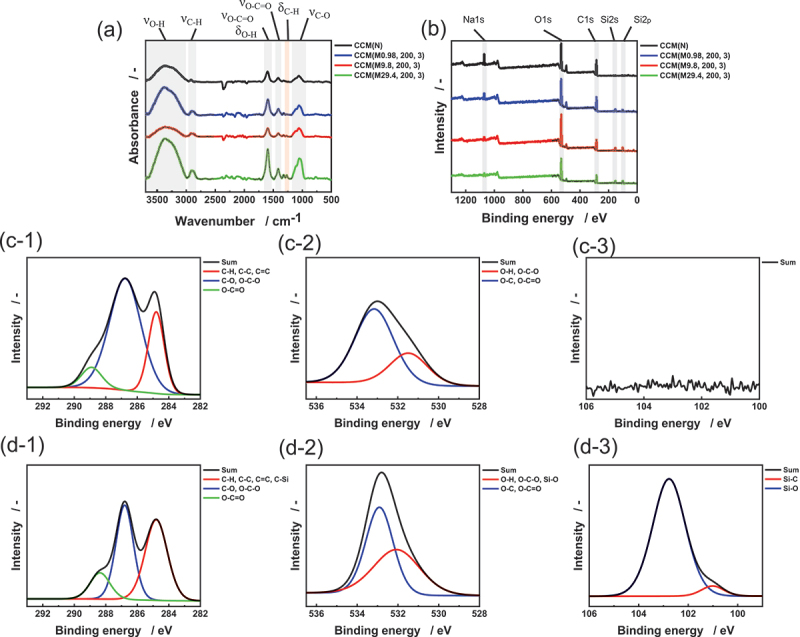
Surface chemical analysis of ultralight CNT/CMC materials. (a) FTIR spectra. (b) Wide scan XPS spectra. Narrow scan XPS spectra of (c) untreated CCM(N) and (d) hydrophobized CCM (M9.8, 200, 3): (c-1, d-1) C 1s, (c-2, d-2) O 1s, and (c-3, d-3) Si 2p orbitals, respectively.

Figure 4 illustrates the surface chemical analysis of ultralight CNT/CMC materials. The FTIR spectrum of CCM (N) (untreated sample) shown in Figure 4(a) exhibits characteristic peaks of CMC. These include O-H stretching (3650 – 3100 cm^−1^), C-H stretching (3000 – 2840 cm^−1^), asymmetric O-C = O stretching and O-H bending from water (1700 – 1550 cm^−1^), symmetric O-C = O stretching (1450 – 1300 cm^−1^), and C-O stretching (1200 – 1000 cm^−1^) [26–28]. In contrast, the hydrophobized samples display an additional peak corresponding to C-H bending from -CH_3_ groups in the range of 1300 – 1250 cm^−1^ [19].

Figure 4(b) illustrates the wide scan XPS spectra of the materials. CCM (N) shows peaks for C 1s (285 eV), O 1s (531 eV), and Na 1 s (1072 eV). The hydrophobized samples exhibit additional peaks for Si 2p (99 eV) and Si 2s (151 eV), indicating the presence of silicon-containing groups on the surface. The Si concentration increases with the amount of MTMS used in the reaction (Table 2).

**Table 2.** Atomic concentrations of elements on sample surfaces.

Sample name	Atomic concentration (at%)			
	C	O	Na	Si
CCM(N)	55.8	37.1	5.6	0
CCM (M0.98, 200, 3)	54.0	37.7	3.2	4.9
CCM (M9.8, 200, 3)	44.8	43.6	2.2	9.5
CCM (M29.4, 200, 3)	43.3	42.5	2.7	11.5

Figure 4 (c-1), (c-2), and (c-3) present the narrow scan XPS spectra for C 1s, O 1s, and Si 2p orbitals of CCM(N), respectively. The C 1s spectrum reveals multiple peaks: C = C from CNT at 284.8 eV, C-H and C-C from CMC, C-O and O-C-O from CMC at 286.8 eV, and O-C = O at 288.9 eV. The O 1s spectrum shows peaks for O-H and O-C-O from CMC at 531.5 eV, and O-C and O-C = O at 533.2 eV [28–32]. In contrast, Figure 4(d-1),(d-2), and (d-3) display the corresponding spectra for CCM (M9.8, 200, 3). The C 1s spectrum exhibits an additional C-Si peak. Similarly, the O 1s spectrum shows a new Si-O peak. The Si 2p spectrum reveals peaks for Si-C at 101.5 eV and Si-O at 103.0 eV, confirming the presence of silicon-containing groups after MTMS treatment [28–32]. In contrast, Figure 4(d-1),(d-2), and (d-3) display the corresponding spectra for CCM (M9.8, 200, 3). The C 1s spectrum exhibits an additional C-Si peak. Similarly, the O 1s spectrum shows a new Si-O peak. The Si 2p spectrum reveals peaks for Si-C at 101.5 eV and Si-O at 103.0 eV, confirming the presence of silicon-containing groups after MTMS treatment [33,34].

The contact angles (see Figure S2 for detailed measurement method) of the samples varied significantly by the surface treatment conditions: CCM (N) could not be measured due to its highly hydrophilic nature, while CCM(M0.98, 200, 3), CCM(M9.8, 200, 3), and CCM(M29.4, 200, 3) exhibited contact angles of 109 ± 5.3°, 121 ± 4.2°, and 113 ± 2.9°, respectively (Table 3). The water droplet contact angle depends on the surface microstructure and the chemical state of the surface, such as functional groups and composition. However, as shown in Figures 2(a) and 3(a), the surface microstructure did not change significantly even after hydrophobization. Therefore, the difference in water droplet contact angle depends on the chemical state of the surface.

**Table 3.** Contact angle and *S_O-H_/S_O-C=_*_*O*_ ratios for each sample surface.

Sample name	Contact angle (°)	*S*_*O-H*_*/S*_*O-C = O*_
CCM(N)	Not measurable	37
CCM (M0.98, 200, 3)	109 ± 5	27
CCM (M9.8, 200, 3)	121 ± 4	20
CCM (M29.4, 200, 3)	113 ± 3	26

To evaluate the relative abundance of hydroxyl groups in these samples, we analyzed the FTIR spectra shown in Figure 4(a). Given the complexity of quantifying hydroxyl groups directly from FTIR spectra due to overlapping moisture peaks, we adopted a comparative approach. We calculated the ratio of the O-H stretching peak area *(S*_*O-H*_, 3650 – 3100 cm^−1^) to the area of the symmetric stretching peak of O-C=O (*S*_*O-C = O*_, 1400 – 1350 cm^−1^). The O-C=O peak, present only in CMC and unaffected by hydrophobization, served as a reference. Table 3 presents the *S*_*O-H*_*/S*_*O-C=O*_ values for each sample. CCM(N) exhibited the highest ratio, consistent with its highly hydrophilic surface that prevented contact angle measurement. CCM (M0.98, 200, 3) showed a higher ratio than CCM (M9.8, 200, 3), indicating insufficient hydrophobization due to the limited amount of MTMS used. Interestingly, CCM (M29.4, 200, 3) displayed a higher *S*_*O-H*_*/S*_*O-C=O*_ ratio than CCM (M9.8, 200, 3), despite the increased MTMS treatment. This observation, supported by the higher Si concentration from XPS results (Table 2), suggests that excess MTMS may physically adsorb on CMC. The adsorbed MTMS can form silanol groups (Si-OH) through hydrolysis, increasing the surface hydroxyl content.

Consequently, CCM (M9.8, 200, 3) demonstrated the optimal balance, exhibiting the lowest amount of hydrophilic components and the highest contact angle among the MTMS-treated samples. This finding highlights the importance of precise control over the hydrophobization process to achieve the desired surface properties in ultralight CNT/CMC materials.

### 3.3. Reversible compressibility

**Figure 5.**
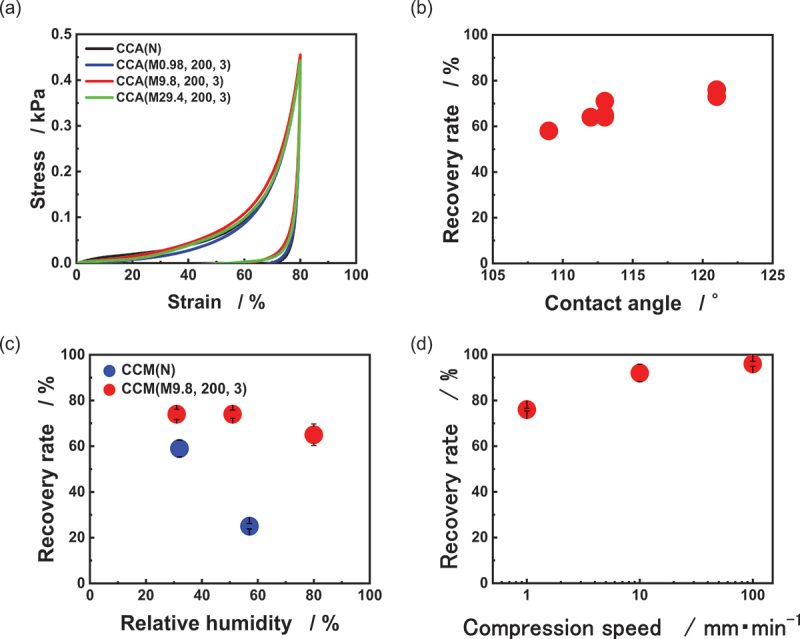
Mechanical properties of hydrophobized ultralight CNT/CMC materials. (a) Stress-strain curves (b) contact angle vs. recovery rate (c) relative humidity vs. recovery rate (d) compression speed vs. recovery rate.

We evaluated the mechanical property of the hydrophobized samples. Figure 5(a) presents the stress–strain curves, which exhibit similar behavior to that of the untreated sample shown in Figure 2(d), with no distinct yield point observed. Figure 5(b) shows the relationship between water contact angle and recovery rate. As the contact angle increased, the recovery rate improved correspondingly, reaching a maximum of 76 ± 3.4% for CCM (M9.8, 200, 3). Detailed numerical data are provided in Table S1 in the Supporting Information.

Figure 5(c) demonstrates the humidity-dependent behavior of the samples. For CCM(N), the recovery rate was 59 ± 3.7% at 32% RH, but decreased significantly to 25 ± 1.2% at 57% RH during compression testing. At 80% RH, the material contracted substantially and could not maintain its shape, preventing compression testing. In contrast, CCM (M9.8, 200, 3) exhibited more stable behavior across humidity levels. It showed recovery rates of 74 ± 2.2% at 31% RH, 74 ± 1.8% at 51% RH, and 65 ± 4.7% at 80% RH. Although there was a slight decrease in recovery rate at 80% RH compared to lower humidity levels, the sample maintained its shape, allowing for compression testing even at high humidity.

Figure 5(d) presents the relationship between compression speed and recovery rate for CCM (M9.8, 200, 3). The recovery rates were 76 ± 0.6% at 1 mm/min, 92 ± 3.8% at 10 mm/min, and 96 ± 1.1% at 100 mm/min. This indicates that faster compression speeds resulted in higher recovery rates. These results demonstrate that the hydrophobized ultralight materials maintain high recovery rates even under high humidity conditions, showcasing improved performance compared to the sample before hydrophobization. CCM (N) contains abundant hydroxyl groups derived from CMC, which readily adsorb moisture from the surrounding air. As humidity increases, more water molecules are absorbed, enhancing water-mediated adhesion between the internal pore walls during compression. This increased adhesive force leads to a reduction in recovery rate. In contrast, hydrophobic modification with MTMS replaces surface hydroxyl groups, suppressing water adsorption and thereby weakening the intermolecular attraction during compression. Although the water contact angle can be influenced by both surface microstructure and chemical composition, Figures 2(a) and 3(a) indicate that the microstructure remained essentially unchanged after hydrophobic treatment. Thus, the variation in contact angle arises primarily from differences in surface chemical state.

To further elucidate the influence of surface intermolecular interaction on the recovery property, we conducted additional experiments using FAS13. Table 4 summarizes the bulk density and recovery rate of CCM(F0.98, 200, 3) prepared for comparison. FAS13, which possesses fluorinated alkyl chains, is known to form highly hydrophobic surfaces and to induce strong intermolecular interactions through polarized C – F bonds. The FAS13-coated sample exhibited a lower recovery rate than CCM(M0.98, 200, 3) (see Supporting Information, Table S2).

**Table 4.** Bulk density and recovery rate of CCM (F0.98, 200, 3).

Sample name	Bulk density (mg/cm^3^)	Recovery rate (%)
CCM (F0.98, 200, 3)	2.0 ± 0.19	36 ± 3.2

This reduction arises from two factors: (i) the increase in bulk density due to the FAS13 coating and (ii) the strong van der Waals interactions associated with the C – F bonds present in FAS13. While a certain density is required for ultralight CNT/CMC materials to achieve elastic recovery, excessive density increases the rigidity of the pore walls, promoting buckling during compression [12]. Moreover, the C – F bond differs from the C – H bond in that it is highly polarized and possesses a permanent dipole moment, which enhances dipole–dipole interactions between adjacent pore walls. According to ref. [35] the total van der Waals coefficient for C–H bonds is 6.06 × 10^−79^ J m^6^, whereas that for C – F bonds reaches 67.0 × 10^−79^ J m^6^ —approximately eleven times larger (Table 5). This substantial increase in intermolecular attraction amplifies adhesion between the internal walls under compression, thereby impeding recovery. In contrast, CCM(M0.98, 200, 3), which predominantly contains C – H bonds, exhibits weaker intermolecular interactions and consequently higher recovery performance.

**Table 5.** Van der Waals forces acting between C-H bonds and C-F bonds.

Chemical bond	Orientation effect (J m^6^)	Induction effect (J m^6^)	Dispersion effect (J m^6^)	Total effect (J m^6^)
C-H	0.422 × 10^−79^ (6.96%)	0.209 × 10^−79^ (3.44%)	5.43 × 10^−79^ (89.6%)	6.06 × 10^−79^
C-F	61.5 × 10^−79^ (91.8%)	2.14 × 10^−79^ (3.20%)	3.37 × 10^−79^ (5.03%)	67.0 × 10^−79^

## 4. Conclusions

In this study, we fabricated ultralight CNT/CMC materials and performed hydrophobic treatment using silane coupling agents to investigate their effect on reversible compressibility. CMC effectively disperses CNTs in water and enables the formation of an ultralight yet mechanically robust framework through the freeze-drying process. However, due to the intrinsic hygroscopic nature of CMC, the compressive recovery of the unmodified CNT/CMC framework remains limited. We therefore examined the reaction conditions of silane coupling agents and investigated how they affect the microstructure and surface state of the ultralight CNT/CMC materials. Evaluations of the surface state using EDX, FTIR, and XPS revealed that the surface of the ultralight material is uniformly coated by a silicon-containing layer derived from the silane coupling agent. As a result, the material surface became hydrophobic and exhibited high reversible compressibility even under high humidity conditions at 80% relative humidity, with a recovery rate of 65%.

From the observation of the FAS13 coating, it was found that the formation of highly polarized C–F bonds increases the intermolecular attraction between pore walls, thereby reducing recovery. This comparison clarifies that excessive surface bonding strength, by strongly polarized functional groups, can hinder structural recovery even though hydrophobicity is achieved. The results indicate that hydrophobization with low-polarity groups such as C–H is key to achieving high recovery performance, as it reduces water adsorption without strengthening intermolecular adhesion. Therefore, constructing an elastic CNT/CMC framework together with appropriately tuned surface hydrophobization offers a rational design pathway toward ultralight materials with stable and reversible mechanical performance.

## Supplementary Material

Supplemental Material
