# Supplementary material for: Improvement of reversible compressibility of ultralight carbon nanotube/carboxymethyl cellulose materials through hydrophobic surface treatment
Source: Sci Technol Adv Mater. 2025 Nov 6;26(1):2580919. doi: 10.1080/14686996.2025.2580919 (PMC12649775; doi:10.1080/14686996.2025.2580919)
Supplement: Supplemental Material [file TSTA_A_2580919_SM3762.docx]

Supporting Information

**Improvement of reversible compressibility of ultralight carbon nanotube/carboxymethyl cellulose materials through hydrophobic surface treatment**

Reo Yanagi^a**^, Hitomi Shimamura^a**^, Kenta Ono^a^, Junko Hieda^a^, and Tomonaga Ueno^a^*

^a^Department of Chemical Systems Engineering, Graduate School of Engineering, Nagoya University, Nagoya,Japan

*Corresponding author

**These authors contributed equally to this work

email: ueno.tomonaga@material.nagoya-u.ac.jp

Graduate School of Engineering, Nagoya University, Furo-cho, Chikusa-ku, Nagoya 464-8603, Japan


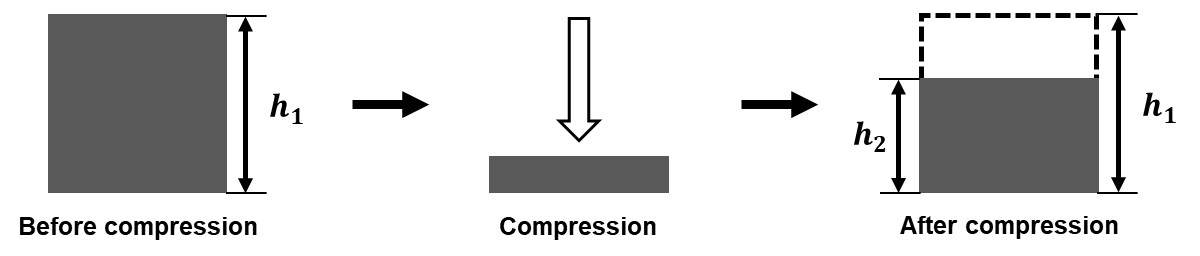


Fig. S1. Schematic illustration of the recovery rate calculation for ultralight CNT/CMC materials after compression. The recovery rate is calculated as $\left( h_{2}/h_{1} \right) \times100$, where $h_{1}$ is the initial height of the sample before compression and $h_{2}$ is the height of the sample after compression and subsequent release.

Figure S2: Contact angle measurement for samples. The contact angle ($\theta$) is calculated using the equation: $\theta=2\tan^{-1} (h/r)$, where $h$ is the height of the droplet from the plane to its apex, and $r$ is the radius of the contact area between the droplet and the sample surface.


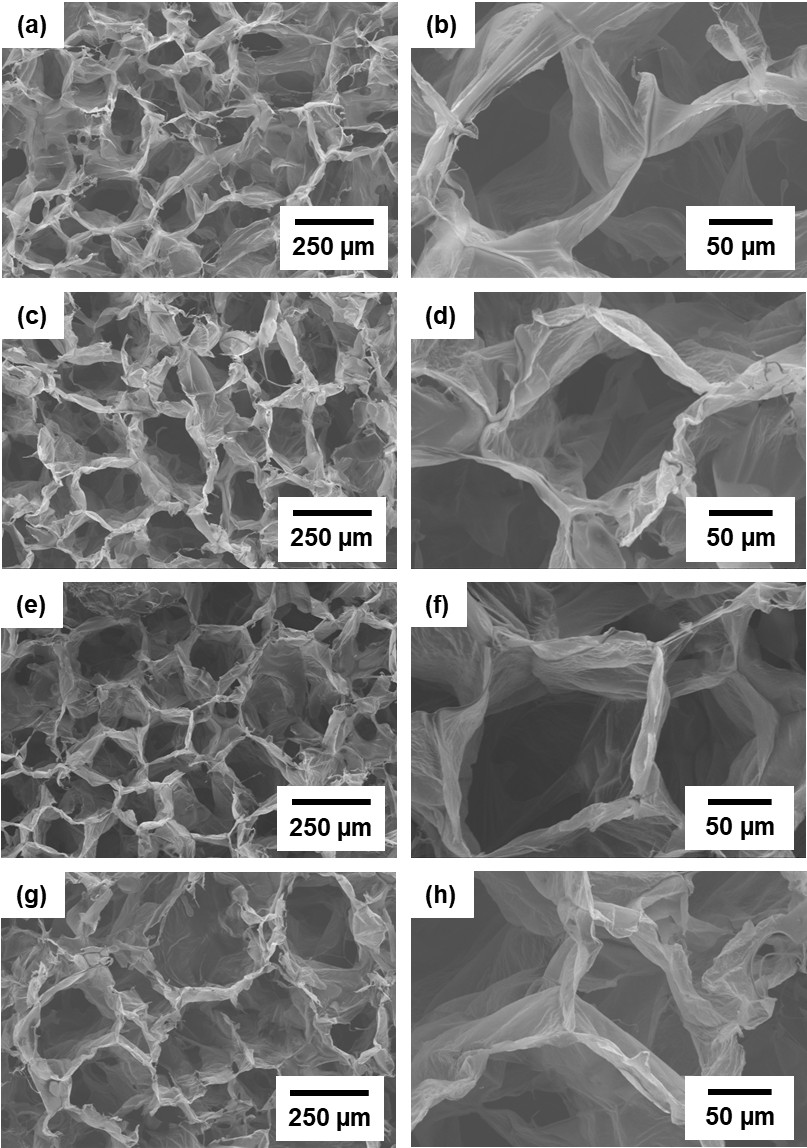


Figure S3: SEM images of the top surfaces of various aerogel samples at different magnifications. (a), (b) CCM(N); (c), (d) CCM(M0.98, 200, 3); (e), (f) CCM(M9.8, 200, 3); (g), (h) CCM(M29.4, 200, 3).

Table S1. Contact angle, *S_O-H_/S_O-C=O_* ratios and recovery rate for each sample surface

| Sample name | Contact angle (°) | *S_O-H_/S_O-C=O_* | Recovery rate (%) |
| --- | --- | --- | --- |
| CCM (M0.98, 200, 3) | 109 ± 5 | 27 | 58 ± 1.8 |
| CCM (M9.8, 150, 3) | 113 ± 2 | 27 | 65 ± 4.8 |
| CCM (M9.8, 175, 3) | 113 ± 5 | 23 | 64 ± 1.2 |
| CCM (M9.8, 200, 1) | 112 ± 6 | 29 | 64 ± 2.3 |
| CCM (M9.8, 200, 3) | 121 ± 4 | 20 | 76 ± 3.4 |
| CCM (M9.8, 200, 5) | 121 ± 3 | 22 | 73 ± 0.4 |
| CCM (M29.4, 200, 3) | 113 ± 3 | 26 | 71 ± 2.9 |

In silane coupling reactions, the hydroxyl groups on the material surface are replaced by the silane coupling agent. Therefore, altering the hydrophobic conditions can change the amount of hydroxyl groups and the amount of adsorbed moisture, potentially affecting the surface's chemical state.

As shown in Table S1, hydrophobization was performed by varying the heating temperature: CCM(M9.8, 150, 3), CCM(M9.8, 175, 3), CCM(M9.8, 200, 3), increasing the heating temperature tends to decrease the value of *S_O-H_/S_O-C=O_*. This indicates that higher heating temperatures remove more adsorbed water internally, reduce surface hydrophilic components, exhibit the maximum water contact angle, and demonstrate a high recovery rate.

Hydrophobization was performed by varying the heating time: CCM(M9.8, 200, 1), CCM(M9.8, 200, 3), CCM(M9.8, 200, 5) show that a shorter heating time hinders sufficient progression of the silane coupling reaction. Consequently, *S_O-H_/S_O-C=O_* and the water contact angle are smaller, indicating a low recovery rate.

Table S2. Density and recovery rate of each sample

| Sample name | Bulk density (mg/cm^3^) | Recovery rate (%) |
| --- | --- | --- |
| CCM (F0.49 , 150, 3) | 1.7 ± 0.03 | 44 ± 3.2 |
| CCM (F0.98, 200, 3) | 2.0 ± 0.19 | 36 ± 3.2 |
| CCM (F9.8, 200, 3) | 4.9 ± 0.53 | 26 ± 3.6 |

Table S2. Shows that the recovery rates of CCM (F0.49, 150, 3), CCM (F0.98, 200, 3), and CCM (F9.8, 200, 3) treated with FAS13 were lower than those treated with MTMS. Thus, the recovery rate decreased with increasing FAS13 amount. The presence of C-F bonds significantly enhanced van der Waals interaction. Under compression, these amplified intermolecular attractions increased adhesive forces and impaired recovery behaviour.
